# Visual Detection of Duck Tembusu Virus With CRISPR/Cas13: A Sensitive and Specific Point-of-Care Detection

**DOI:** 10.3389/fcimb.2022.848365

**Published:** 2022-02-17

**Authors:** Dongdong Yin, Lei Yin, Jieru Wang, Xuehuai Shen, Xiaocheng Pan, Hongyan Hou, Ruihong Zhao, Xiaomiao Hu, Guijun Wang, Kezong Qi, Yin Dai

**Affiliations:** ^1^ Institute of Animal Husbandry and Veterinary Science, Anhui Academy of Agricultural Sciences, Livestock and Poultry Epidemic Diseases Research Center of Anhui Province, Anhui Province Key Laboratory of Livestock and Poultry Product Safety Engineering, Hefei, China; ^2^ Anhui Province Key Laboratory of Veterinary Pathobiology and Disease Control College of Animal Science and Technology, Anhui Agricultural University, Hefei, China

**Keywords:** duck tembusu virus, RPA, CRISPR/Cas13a, lateral flow detection, fluorescence detection

## Abstract

Duck tembusu virus (DTMUV), which causes huge economic losses for the poultry industries in Southeast Asia and China, was first identified in 2010. DTMUV disease has become an important disease that endangers the duck industry. A sensitive, accurate, and convenient DTMUV detection method is an important means to reduce the occurrence of the disease. In this study, a CRISPR/Cas13a system was combined with recombinase polymerase amplification to develop a convenient diagnostic method to detect DTMUV. The novel method was based on isothermal detection at 37°C, and the detection was used for visual readout or real-time analysis. The assay was highly sensitive and specific, with a detection limit of 1 copy/μL of the target gene and showed no cross-reactivity with other pathogens. The enhanced Cas13a detection worked well with clinical samples. Overall, a visual, sensitive, and specific nucleic acid detection method based on CRISPR/Cas13a proved to be a powerful tool for detecting DTMUV.

## Introduction

In April 2010, a new epidemic disease erupted in breeding ducks and laying ducks in Fujian, Hebei, Zhejiang, Shandong, and other provinces in China. The disease is characterized by a sharp loss of appetite and a significant decline in egg production, causing huge economic losses to the duck industry (Cao et al., 2011; Li et al., 2013). In-depth study of its etiology revealed that the disease is caused by duck tembusu virus (DTMUV). For ducks with high egg production rates, the egg production rate drops rapidly to 20%–30% 4–5 days after disease onset, and production stops about 7 days after disease onset (Homonnay et al., 2014; Thontiravong et al., 2015). The disease has become an important disease that endangers the poultry industry (Chen et al., 2014; Dai et al., 2015; Ti et al., 2015; Lu et al., 2016; Peng et al., 2020). For the prevention and treatment of the virus, it is necessary to detect the disease as early as possible and take as many measures as possible to prevent the spread of the virus; therefore, the establishment of a sensitive, accurate, and convenient DTMUV detection method is an important means to reduce the occurrence of DTMUV disease.

At present, the detection of DTMUV is usually based on reverse transcription PCR (RT-PCR), real-time RT-PCR, loop-mediated isothermal amplification (LAMP), and virus isolation (Wang et al., 2011; Jiang et al., 2012; Tang et al., 2012; Yan et al., 2012). These methods are time consuming, labor-intensive, and complicated to operate, and rapid and accurate on-site detection of the virus remains difficult. According to the standards of the World Health Organization, ideal pathogen diagnostic tests are cheap, sensitive, specific, easy to use, and fast, and they can be realized without large-scale equipment (Kosack et al., 2017). Point-of-care testing (POCT) helps to improve efficiency, optimize decision making in a timely manner, and reduce costs, especially in resource-constrained areas (Hansen, 2015; Alp, 2018).

Clustered regularly interspaced short palindromic repeats (CRISPR)/CRISPR-associated protein 13a (CRISPR/Cas13a) is a new type of CRISPR system that only targets RNA. It can specifically cleave single-stranded target RNA under the guidance of CRISPR RNA (crRNA) and has collateral cleavage activity (East-Seletsky et al., 2016; Gootenberg et al., 2017). Zhang and colleagues (Kellner et al., 2019) have developed a diagnostic system called SHERLOCK (specific high-sensitivity enzymatic reporter unlocking) based on CRISPR/Cas13a, which can be used to quickly and easily detect a small amount of RNA in clinical samples. Specifically, the SHERLOCK system is an isothermal amplification system that combines recombinase polymerase amplification (RPA), CRISPR/Cas13a, crRNA, and fluorescent reporter molecules. SHERLOCK can achieve visual readings by combining with lateral flow readout, eliminating the dependence on thermal cyclers (Gootenberg et al., 2018). In recent years, CRISPR/Cas13a-based detection has been successfully applied to detect porcine reproductive and respiratory syndrome virus (PRRSV), Avian influenza A (H7N9) virus, and Ebola virus (EBOV) (Liu et al., 2019; Qin et al., 2019; Chang et al., 2020). In this study, a Cas13a detection method combined with RPA, T7 transcription, and the collateral effect of CRISPR/Cas13a was developed for sensitive, specific, equipment-free, and visual detection of DTMUV targeting the conserved DTMUV *E* gene.

## Materials and Methods

### Viruses and Clinical Samples

The DTMUV AH-F10 strain (Zhao et al., 2011) isolated in 2010 in our laboratory was used in the present study (Accession number: KM102539.1). Novel duck parvovirus (NDPV), muscovy duck reovirus (MDRV), duck plague virus (DPV), goose astrovirus (GoAstV), and fowl adenovirus serotype 4 (FAdV-4) were isolated in our laboratory. Newcastle disease virus (NDV) inactivated vaccine was produced by Shandong Lvdu Bio-Sciences & Technology Co., Ltd. 15 tissue samples were collected from different farms.

### RPA Primer Design and crRNA Preparation

The RPA primers were selected in the conserved nucleotide region of the *E* gene. The T7 promoter sequence (GAAATTAATACGACTCACTATAGGG) was appended to the 5′ end of the RPA forward primer. For crRNA preparation, the DNA templates of crRNA were appended with the T7 promoter sequence and synthesized as primers by General Biological System (Anhui) Co. (**Table 1**). The FAM-N6-BHQ1 probe used in the fluorescent reporter assays was synthesized by General Biological System (Anhui) Co. Two oligonucleotides were annealed to a double‐stranded DNA by using Annealing Buffer for DNA Oligos (Beyotime, China). The double-stranded DNA was purified by gel extraction. According to the instructions of the HiScribe T7 Quick High Yield RNA Synthesis kit (NEB, USA), the double-stranded DNA was transcribed to crRNA. Finally, crRNA was purified using NucAway™ Spin Columns (Invitrogen, USA) according to the manufacturer’s instructions and stored at −80°C.

**Table 1:** The crRNA, primers, and probes used in this study.

Primers	Sequences(5’-3’)
RPA-F	GAAATTAATACGACTCACTATAGGGGAAGCTGAAAGGAATGACCTACCCGATGT
RPA-R	TGACTGTTATCAAGCGTCCAACTGGTGTC
crRNA-F	GATTTAGACTACCCCAAAAACGAAGGGGACTAAAACCCAAAAACCTGATGAATGCCTTTCCCAA
crRNA-R	TTGGGAAAGGCATTCATCAGGTTTTTGGGTTTTAGTCCCCTTCGTTTTTGGGGTAGTCTAAATC
Probe	FAM-mArArUrGrGrCmAmArArUrGrGrCmA-Bio

### Nucleic Acid Preparation

The viral genomic nucleic acids of the DTMUV AH-F10 strain, NDPV, MDRV, DPV, GoAstV, FAdV-4, and NDV were extracted with a TIANamp Virus DNA/RNA Kit (Tiangen, China) according to the manufacturer’s instructions and stored at −80°C until use.

### Cas13a Nucleic Acid Detection

For RPA, 1 μL of cDNA or DNA was amplified in a 50-μL reaction system for 20 min at 37°C, according to the instructions of the Basic isothermal amplification reagent kit-Powder (Magigen, China). For Cas13a detection with lateral flow detection, the Cas13a reaction system consisted of 50 μL containing 22.5 nM crRNA, 45 nM Cas13a (Magiltd, China), 125 nM FAM-N6-BHQ1 probe, 0.25 μL RNase inhibitor, 2.5 μL NTP Buffer Mix, 0.4 μL T7 RNA Polymerase Mix (NEB, USA), and 1 μL RPA products. Cas13a detection was performed at 37°C for 40 min. The detection products were diluted 10 times with Hybridetect Assay Buffer (Magiltd, China), loaded onto the lateral flow strips (Magigen, China), and placed for 5 min to observe the results. For Cas13a detection with fluorescence detection, the FAM-N6-BHQ1 probe was replaced by the RNA reporter (RNAse Alert v2, Thermo Fisher Scientific, USA) in a 50-μL Cas13a reaction system. Reactions were performed in an ABI StepOnePlus™ (Applied Biosystems, USA) instrument at 37°C for 60 min, and fluorescence intensity kinetics was recorded every 5 min.

### Sensitivity and Specificity of the Cas13a Lateral Flow Detection

The E fragments of DTMUV AH-F10 strain were cloned into the pMD-19T vector. Ten-fold serial dilutions of pMD19T-E (1.0 × 10^8^ to 1.0 × 10^0^ copies/μL) were prepared as a template for Cas13a lateral flow detection, then visually observed. The specificity of lateral flow detection was assessed using the genomic cDNA or DNA of a panel of pathogens, including NDPV, MDRV, DPV, GoAstV, NDV, and FAdV-4.

### Sensitivity and Specificity of the Cas13a Fluorescence Detection

Aliquots of the DTMUV standard DNA ranging from 1.0 × 10^8^ to 1.0 × 10^0^ copies/μL were prepared as a template for Cas13a l fluorescence detection. The fluorescence intensity was read to determine the limit of detection to evaluate the sensitivity. The specificity of fluorescence detection was assessed using the genomic cDNA or DNA of a panel of pathogens, including NDPV, MDRV, DPV, GoAstV, NDV, and FAdV-4.

### Validation With Clinical Samples

A total of 15 ovarian tissue samples were collected from 2018 to 2021 from farms located in Anhui Province where laying ducks had decreased egg production. All samples were used to confirm the applicability of DTMUV-specific lateral flow and fluorescence assays in clinical diagnosis. Then, the results were compared with those obtained with RT-PCR described previously (Liu et al., 2013), which was run in parallel for the above clinical samples. The studies involving animals were reviewed and approved by the Ethics Committee of Anhui Academy of Agricultural Sciences, and the owners of animals provided written informed consent to participate in this study.

## Results

### Validation of the Cas13a Detection

**Figure 1:**
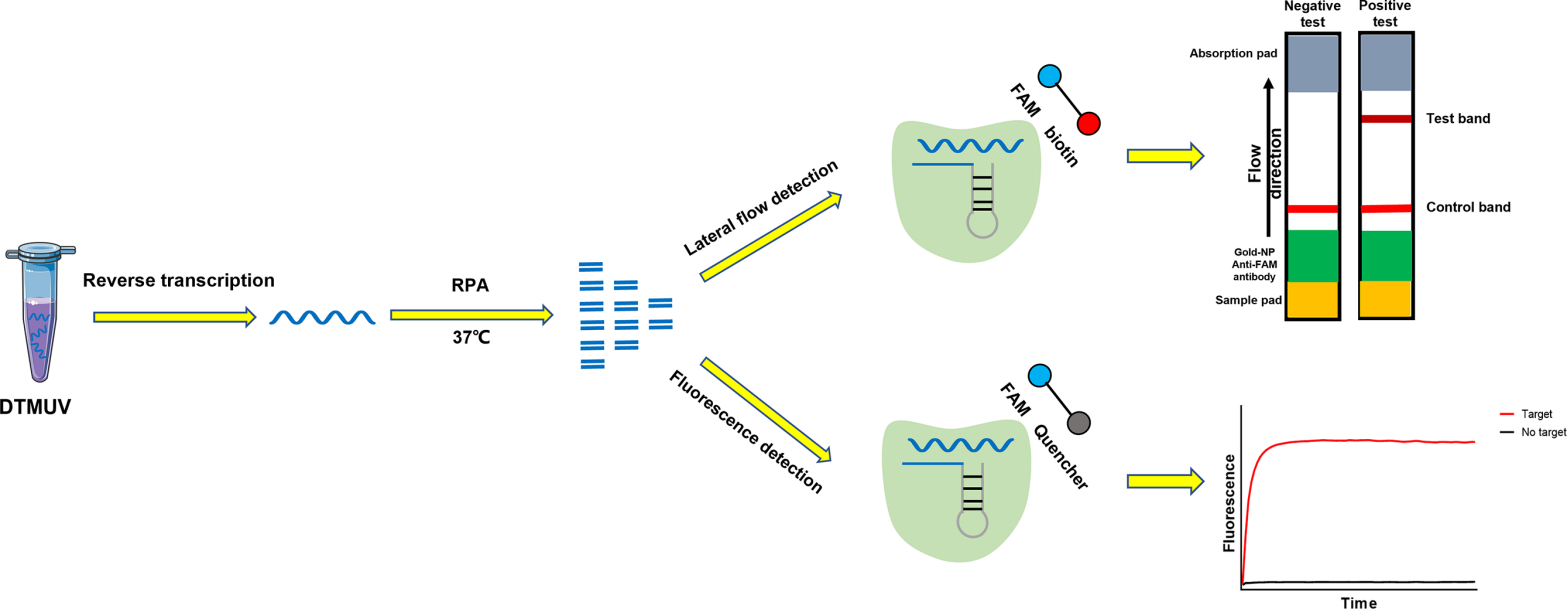
Schematic illustration of the workflow for Cas13a DTMUV testing.

**Figure 2:**
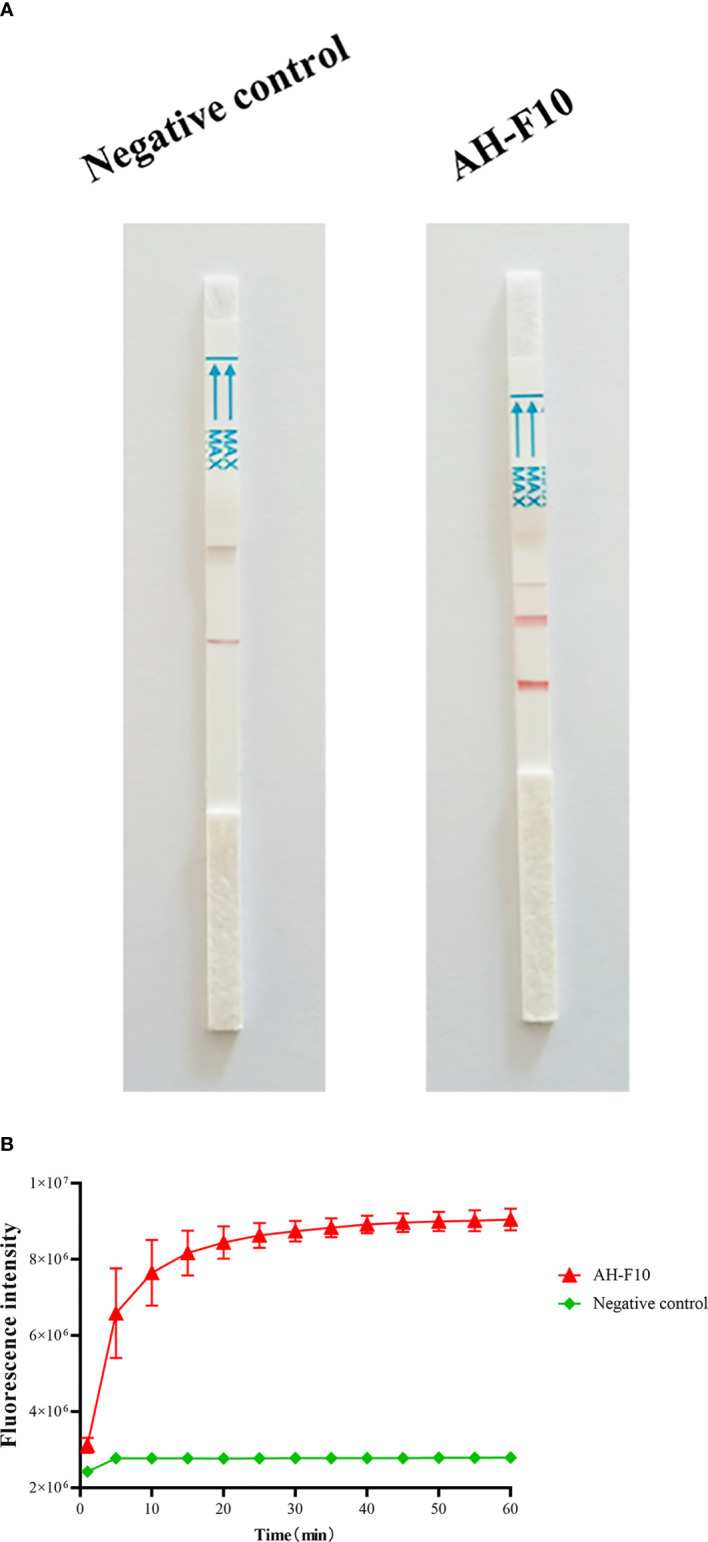
Performance of DTMUV Cas13a lateral flow and fluorescence detection. **(A)** Analysis of DTMUV by Cas13a lateral flow detection. **(B)** Analysis of DTMUV by Cas13a fluorescence detection. *n* = 3 technical replicates; values represent mean ± SEM.

We performed RPA combined with CRISPR/Cas13a to detect DTMUV according to the schematic diagram in **Figure 1**. To verify the effectiveness of the designed primers, we performed Cas13a experiments using the RPA products as templates. The experimental group showed that lateral flow detection strips of DTMUV appeared as obvious positive bands (**Figure 2A**). As shown in **Figure 2B**, the fluorescence units of the positive group increased rapidly with time until the peak value, while the negative group had no value. The results indicated that the Cas13a lateral flow and fluorescence detection could be used to detect DTMUV.

### Specificity and Sensitivity of the Cas13a Lateral Flow Detection

**Figure 3:**
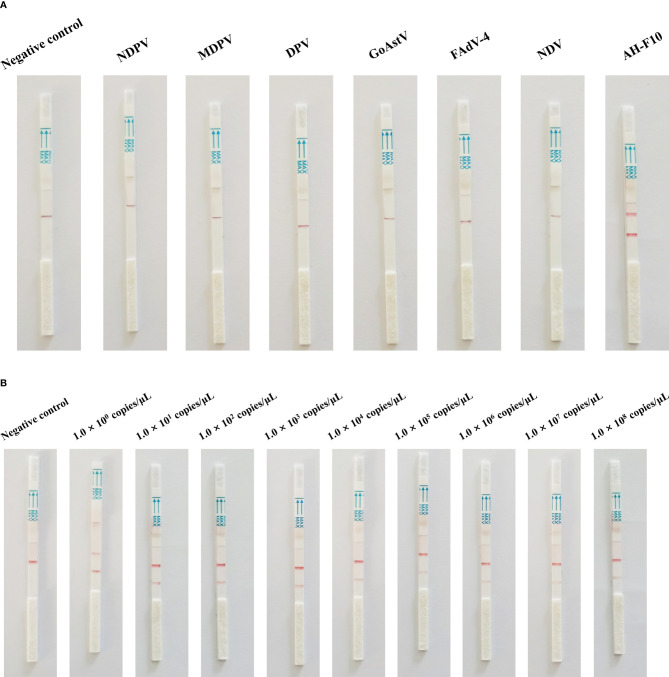
Specificity and sensitivity of Cas13a lateral flow detection. **(A)** Specificity of Cas13a lateral flow detection. **(B)** Sensitivity of Cas13a lateral flow detection.

When the analytical specificity analysis was conducted, the positive band was observed on the lateral flow detection strip of DTMUV, and the lateral flow detection strips of five other viruses had no positive bands (**Figure 3A**). As the analytical sensitivity analysis proceeded, positive bands could be observed in the test line on the strips with 1.0 × 10^8^ to 1.0 × 10^0^ copies of DTMUV standard DNA serving as the template (**Figure 3B**). Thus, the detection limit of the Cas13a lateral flow detection was 1 copy/μL. As mentioned above, these results revealed that the Cas13a lateral flow detection for DTMUV was highly specific and sensitive.

### Specificity and Sensitivity of the Cas13a Fluorescence Detection

**Figure 4:**
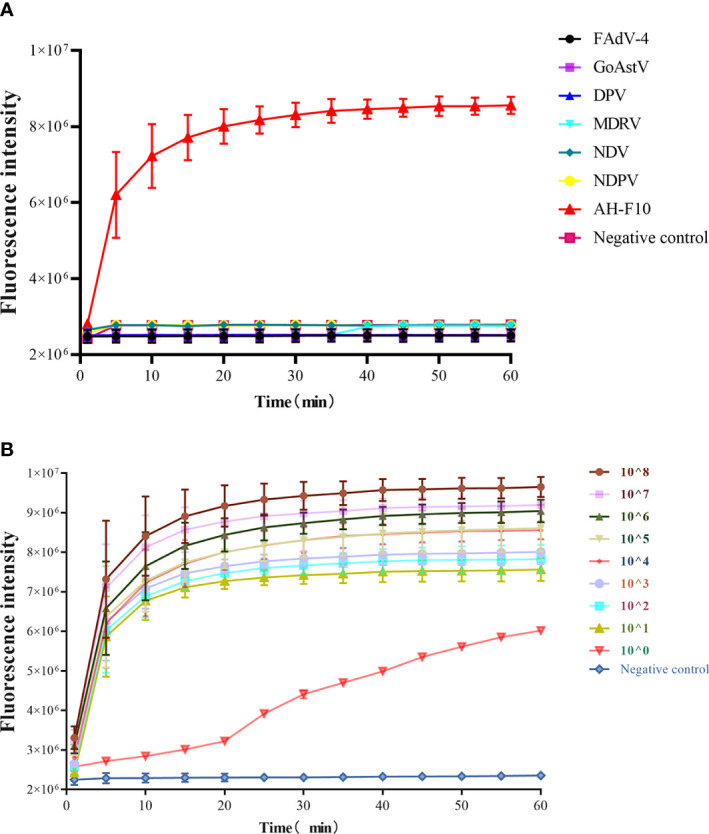
Specificity and sensitivity of Cas13a fluorescence detection. **(A)** Specificity of Cas13a fluorescence detection. **(B)** Sensitivity of Cas13a fluorescence detection. *n* = 3 technical replicates; values represent mean ± SEM.

In the analytical specificity analysis, only DTMUV was amplified with the development of a typical fluorescence curve, and there was no cross-reaction with other viruses (**Figure 4A**), suggesting that Cas13a fluorescence detection could be used for the specific detection of DTMUV. The sensitivity of the Cas13a fluorescence detection was tested with 10‐fold serially diluted template DTMUV standard DNA. As shown in **Figure 4B**, nine orders of magnitude from 1.0 × 10^8^ copies/μL down to 1.0 × 10^0^ copies/μL template could be detected. These data showed that the detection limit of Cas13a fluorescence detection was 1 copy/μL.

### Cas13a Detection in Clinical Samples

To verify the application of Cas13a lateral flow and fluorescence detection in clinical samples, 15 tissue samples collected from different farms were detected by Cas13a lateral flow, fluorescence detection, and RT-qPCR. Six tissue samples were DTMUV-positive, and nine samples were DTMUV-negative, as determined by Cas13a lateral flow detection. Cas13a fluorescence detection and RT-qPCR displayed the same results as Cas13a lateral flow detection in clinical samples (**Table 2**). These data indicated that Cas13a lateral flow and fluorescence detection could be used in clinical samples, and Cas13a lateral flow detection does not require expensive equipment.

**Table 2:** Detection results of clinical samples in Cas13a lateral flow detection, Cas13a fluorescence detection, and RT-qPCR assays.

Assay	Number of samples	
	Positive	Negative
RT-qPCR	6	9
Cas13a lateral flow detection	6	9
Cas13a fluorescence detection	6	9

## Discussion

DTMUV disease was first discovered in parts of southern China in April 2010. DTMUV disease mainly affects ducks in the laying stage (Cao et al., 2011; Li et al., 2013). The disease causes a sharp drop in egg production, resulting in serious economic losses to laying duck farmers, and has a great impact on the breeding industry. Although a DTMUV vaccine has been applied to poultry farms, DTMUV outbreaks still occur in many areas (Lu et al., 2016; Peng et al., 2020; Zhu et al., 2021). Therefore, timely monitoring of DTMUV through diagnosis is essential for early control of the epidemic. At present, most of the available DTMUV detection methods have many shortcomings, such as not being suitable for field detection and low sensitivity. Ideal diagnostic methods are inexpensive, accurate, fast, and easy to operate, and should not require specialized equipment. Therefore, there is an urgent need to develop a specific and sensitive DTMUV detection method with minimal equipment requirements.

In this study, a new method for DTMUV nucleic acid detection based on CRISPR/Cas13a was established, which could detect DTMUV with minimal equipment. In order to determine the appropriate target region, a set of DTMUV genomes were compared and analyzed. According to the comparison results, the RPA primer and crRNA of the DTMUV *E* gene were designed. In Yan et al.’s study, the detection limit of real-time PCR based on a TaqMan probe was 50 copies per reaction (Yan et al., 2012). In Yun et al.’s study, the detection limit of the real-time PCR method based on minor groove binding was 10 copies per reaction (Yun et al., 2012). The RT-PCR method established by Liu et al., (2013) can detect 20 copies per reaction. In addition, the detection limit of the LAMP method established by Tang et al. can reach 10 copies per reaction, and it does not require much equipment (Tang et al., 2012), but it still has shortcomings such as high target selectivity and cross-contamination, which render the method unsuitable for laboratory research. The method established in this study had a low detection limit of 1 copy/μL for DTMUV and was hence more sensitive than the above method.

Cas13a detection can not only be used for the analysis of fluorescence detection samples but can also be combined with lateral flow detection for visual readout, making it available for use both in the laboratory and in the field. At the same time, the combination of RPA and crRNA-specific sequence identification makes Cas13a detection more specific (Gootenberg et al., 2017). In addition, another important advantage is that Cas13a detection is performed at 37°C, which is convenient for use in poorly equipped laboratories or in the field (Gootenberg et al., 2017; Gootenberg et al., 2018; Myhrvold et al., 2018). The Cas13a detection method has been applied to the detection of other pathogens. Myhrvold et al. utilizes the Cas13-based SHERLOCK platform to detect Zika virus (ZIKV) and dengue virus (DENV) in patient samples at concentrations as low as 1 copy per microliter. Chang et al. used this method to detect PRRSV with specificity and sensitivity, and it was successfully applied to detect clinical specimens from different farms. In conclusion, this method provides a new technical means for the molecular detection of pathogens.

In conclusion, a visual and reliable nucleic acid detection method based on CRISPR/Cas13a was established to identify DTMUV. This is the first report using Cas13a methods to detect DTMUV. The novel method had the advantages of high specificity, high sensitivity, and minimal equipment, particularly in resource‐poor settings.

## Data Availability Statement

The original contributions presented in the study are included in the article/supplementary material. Further inquiries can be directed to the corresponding author.

## Author Contributions

DY conceived and designed the experiments. DY, LY, and JW were responsible for sampling and sample testing. HH, XP, and XS analyzed the data. DY, RZ, and XH wrote the paper. GW, KQ, and YD edited the paper. All authors contributed to the article and approved the submitted version.

## Funding

This study was supported financially by the Major Science and Technology Special Project in Anhui Province (No. 202003a06020012), the Anhui Academy of Agricultural Sciences Platform Project (No. 2021YL065), and the Anhui Province Poultry Industry Technology System (No. AHCYJSTX-06).

## Conflict of Interest

The authors declare that the research was conducted in the absence of any commercial or financial relationships that could be construed as a potential conflict of interest.

## Publisher’s Note

All claims expressed in this article are solely those of the authors and do not necessarily represent those of their affiliated organizations, or those of the publisher, the editors and the reviewers. Any product that may be evaluated in this article, or claim that may be made by its manufacturer, is not guaranteed or endorsed by the publisher.
